# Mortality Trends and Demographic Disparities Among Patients With Lymphoid Leukemia and Septicemia

**DOI:** 10.7759/cureus.91920

**Published:** 2025-09-09

**Authors:** Hiral Undhad, Pooja Joshi, Nidhi Patil, Neha Uppal, Simranjeet Bedi

**Affiliations:** 1 Internal Medicine, Jiangsu University School of Medicine, Zhenjiang, CHN; 2 Medicine, Kanti Devi Medical College, Hospital and Research Center, Mathura, IND; 3 Emergency Medicine, Lister Hospital, Stevenage, GBR; 4 Internal Medicine, Integrated Medical Care Hospital, Lahore, PAK; 5 Internal Medicine, Kharkiv National Medical University, Kharkiv, UKR

**Keywords:** age-adjusted mortality rate, cdc mcd, lymphoid leukemia, retrospective study, septicemia

## Abstract

Introduction: Lymphoid leukemia is a major cause of death, and its link with septicemia is progressively studied. It is crucial to understand that the relationship is important for identifying high-risk populations and designing targeted public health interventions.

Aims: This study aims to evaluate mortality trends in lymphoid leukemia with septicemia as a contributing cause of death, using the CDC Multiple Causes of Death (MCD) database.

Methodology: A study retrospectively examined data from the CDC MCD database to determine trends in deaths among individuals 25 years and older in the US from 1999 through 2020. They examined deaths with lymphoid leukemia as the underlying cause of death and septicemia as a contributing cause. The data were categorized by sex, race, geographic region, and location of death. Age-adjusted mortality rate (AAMR) and annual percentage change (APC) were calculated.

Results: From 1999 to 2020, 17,265 adults over 25 died with lymphoid leukemia and septicemia. Most deaths occurred in metropolitan areas (N = 14,267, 82.6%). Males (N = 10,825, 62.7%) and White individuals (N = 14,953, 86.6%) had the highest mortality. The crude death rate was 3.9 per million population.

Conclusion: This study highlights significant mortality trends increasing in lymphoid leukemia with septicemia with disparities by gender and race. Findings underscore the need for targeted prevention strategies and improved healthcare access.

## Introduction

Lymphoid leukemia, also known as lymphocytic leukemia, is characterized by the malignant transformation and uncontrolled proliferation of early-stage lymphoid cells, which eventually differentiate into either B or T lymphocytes [1]. In developed countries, chronic lymphocytic leukemia (CLL) has the highest incidence among adult leukemias [2]. Data collected from the CDC Wide-Ranging Online Data for Epidemiologic Research (WONDER) database from 1999 to 2022 in the United States showed that overall mortality rates for lymphoid leukemia declined, but they began to rise again between 2018 and 2022. Men had higher age-adjusted mortality rates (AAMRs) compared to women. Between 1999 and 2019, patients with lymphoid leukemia who were over the age of 85 exhibited a higher crude mortality rate. The Midwest region experienced the highest mortality rates from lymphoid leukemia among all U.S. regions, and White individuals had the highest AAMRs for the condition among all racial groups [3].

Septicemia is a life-threatening bloodstream infection. Multiple studies have demonstrated that mortality rates significantly increase in patients with septicemia who have underlying hematologic malignancies, including lymphoid leukemias [4]. Hematologic malignancies and their associated therapies elevate the risk of developing sepsis, with up to 30% of patients diagnosed within the first year. Among these patients, AAMRs were highest in non-Hispanic Black males aged 65 to 74 years [5].

Infectious complications are a leading contributor to morbidity and mortality in patients with CLL, largely due to humoral immune deficiency associated with the disease itself and the additional immunosuppressive effects of treatment [6]. Chemotherapy weakens the immune system by suppressing bone marrow, causing neutropenia, which promotes bacterial invasion. CLL patients are especially prone to septicemia, a leading cause of death in this group [2]. Although numerous studies have independently examined the impact of lymphoid leukemia and septicemia on mortality, few have explored how the co-occurrence of these conditions influences overall death rates. Due to the limited literature on this topic, it is essential to analyze the relationship between lymphoid leukemia and septicemia as contributors to mortality using the CDC Multiple Causes of Death (MCD) database.

The objective of this study is to evaluate mortality trends in lymphoid leukemia with septicemia as a contributing cause of death, using the CDC MCD database from 1999 to 2020, stratified by sex, race, and geographic region.

## Materials and methods

A retrospective analysis was conducted using the CDC WONDER MCD database [7]. This publicly available database contains deidentified information from U.S. death certificates, and therefore, the study was classified as non-human participant research, exempt from institutional review board (IRB) approval [8]. Data extraction was performed on July 18, 2025.

Case definitions and study population

We included deaths recorded between 1999 and 2020 among individuals aged 25 years and older. Lymphoid leukemia (International Classification of Diseases (ICD-10) ICD-10: C91) was selected as the underlying cause of death, and septicemia (ICD-10: A41) was included as a multiple cause of death [9]. The ≥25 years cutoff was used to ensure methodological consistency with prior national studies and to reduce variability associated with the very small number of deaths in younger adults.

Query parameters and data extraction

CDC WONDER provides preset options for stratification. For this study, data were grouped by sex, race/ethnicity (American Indian or Alaska Native, Asian or Pacific Islander, Black or African American, or White individuals), geographic region/urbanization (2013 National Center for Health Statistics (NCHS) urban-rural classification scheme: large central metro, large fringe metro, medium metro, small metro, micropolitan, and non-core), and place of death (inpatient hospital, outpatient facility, home, hospice, or nursing home). Stratified data were obtained through separate queries. Suppressed data (counts < 10) were excluded from subgroup analyses and not estimated.

Mortality rates

Crude death rates and AAMRs were directly calculated by the CDC WONDER system using the annual U.S. population aged ≥25 years as the denominator and standardized to the 2000 U.S. Standard Population. Rates are expressed per 1,000,000 population.

Statistical analysis

Temporal trends in AAMRs were evaluated using Joinpoint Regression Analysis (Joinpoint Software Version 5.3.0.0, released November 2024, Statistical Methodology and Applications Branch, Surveillance Research Program, National Cancer Institute (NCI), USA). The maximum number of joinpoints allowed was set at three, and model selection was performed using permutation tests with a significance level of α = 0.05. Annual percentage change (APC) values were calculated for each segment of the trend, with statistically significant inflection points reported. No adjustments for multiple comparisons were applied, consistent with prior national database analyses.

## Results

From 1999 to 2020, the CDC MCD database recorded 17,265 deaths in the United States among individuals aged 25 years and older. Among these, deaths where lymphoid leukemia (ICD-10: C91) was listed as the underlying cause of death and septicemia (ICD-10: A41) was recorded as a multiple cause of death were included in the study (17,265). The crude mortality rate for lymphoid leukemia with septicemia as a contributing cause was 3.9 per 1,000,000 population. Deaths due to causes other than these criteria were excluded.

Demographic characteristics

Among the total deaths analyzed, males accounted for 10,825 (62.7%), while females accounted for 6,440 (37.3%). The mortality rate for lymphoid leukemia with septicemia as a contributing cause was higher in males compared to females, indicating a potential demographic disparity. Regarding racial distribution, the largest proportion of deaths occurred among White individuals (n = 14,953, 86.6%), followed by Black or African American individuals (n = 1,949, 11.3%), Asian or Pacific Islander individuals (n = 295, 1.7%), and American Indian or Alaska Native individuals (n = 68, 0.4%). The mortality burden was highest among White individuals, highlighting racial disparities in mortality trends related to melanoma and cardiovascular disease.

Geographic characteristics

A majority of deaths occurred in metropolitan areas (n = 14,267, 82.6%), while non-metropolitan areas accounted for n = 2998, 17.4% of deaths. Regarding the place of death, most deaths occurred in medical facilities as inpatients (n = 14,953, 86.6%), followed by nursing homes (n = 660, 3.8%), decedents' homes (n = 631, 3.7%), hospice facilities (n = 425, 2.5%), and outpatient medical facilities (n = 375, 2.2%).

Temporal trends

**Figure 1:**
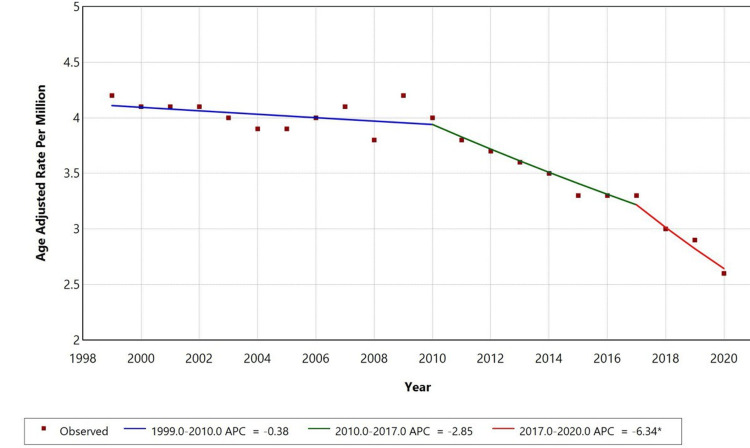
Overall age-adjusted mortality rates among adults aged 25+ in the United States, 1999–2020. * Indicates that the annual percentage change (APC) is significantly different from zero at alpha = 0.05 level.

From 1999 to 2020, the AAMR for lymphoid leukemia with septicemia in adults aged 25 and older in the United States showed a consistent decline. Between 1999 and 2010, the AAMR decreased slowly with an APC of -0.38% (p < 0.05). A more pronounced decline was observed from 2010 to 2017, with an APC of -2.85% (p < 0.05). The most significant drop occurred between 2017 and 2020, during which the AAMR declined sharply with an APC of -6.34% (p < 0.05). Significant inflection points were observed in 2010 and 2017, indicating potential impacts of treatment advances, improved infection control, or broader public health interventions on mortality patterns, as shown in Figure 1.

**Figure 2:**
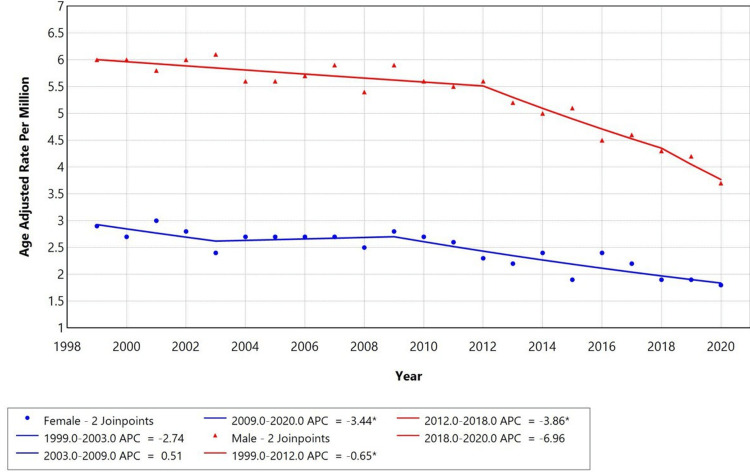
Trends in sex-stratified age-adjusted mortality rates among adults aged 25+ in the United States, 1999–2020. * Indicates that the annual percentage change (APC) is significantly different from zero at alpha = 0.05 level.

When stratified by gender, distinct temporal trends in AAMR were observed among adults aged 25 and older in the United States from 1999 to 2020. For females, the AAMR remained relatively stable in the early years, with a modest decline from 1999 to 2003 (APC: -2.74%), followed by a slight increase between 2003 and 2009 (APC: +0.51%). A sustained and significant decline was observed from 2009 to 2020 (APC: -3.44%, p < 0.05). For males, the overall AAMR was consistently higher than for females throughout the study period. Between 1999 and 2012, the AAMR declined modestly (APC: -0.65%, p < 0.05), followed by a steeper and statistically significant decline from 2012 to 2018 (APC: -3.86%, p < 0.05), and an even sharper decrease between 2018 and 2020 (APC: -6.96%) as represented in Figure 2.

**Figure 3:**
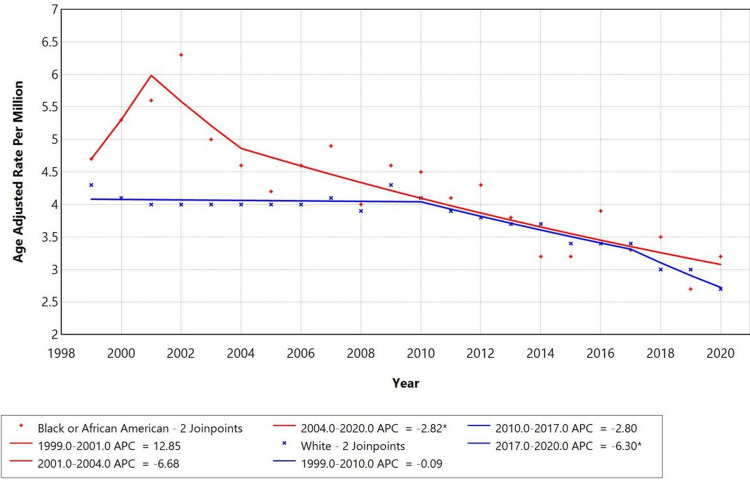
Trends in age-adjusted mortality rates stratified by race among adults aged 25+ years in the United States, 1999 to 2020. * Indicates that the annual percentage change (APC) is significantly different from zero at alpha = 0.05 level. Temporal trends for American Indian or Alaska Native and Asian or Pacific Islander individuals are not displayed due to data suppression for counts < 10, limiting reliable trend analysis.

Racial disparities were observed in lymphoid leukemia with septicemia as a contributing cause trend. Black or African American individuals had the highest AAMR, peaking around 2001 at approximately 6.0 per million, followed by White individuals whose AAMR remained relatively stable around 4.1 per million until 2010, before beginning a gradual decline. For Black or African American individuals, the AAMR increased sharply from 1999 to 2001 (APC: +12.85%), then declined from 2001 to 2004 (APC: -6.68%), followed by a sustained significant decrease from 2004 to 2020 (APC: -2.82%, p < 0.05). In comparison, White individuals showed a near-stable trend from 1999 to 2010 (APC: -0.09%), followed by a steeper decline from 2010 to 2017 (APC: -2.80%), and an accelerated drop from 2017 to 2020 (APC: -6.30%, p < 0.05), as shown in Figure 3. Trends for other racial groups were not displayed due to data suppression for counts < 10, limiting reliable trend analysis.

## Discussion

This retrospective study utilized the CDC MCD database to analyze mortality trends for lymphoid leukemia (ICD-10: C91) with septicemia (ICD-10: A41) as a contributing cause in the United States from 1999 to 2020 among individuals aged 25 years and older. A total of 17,265 deaths were identified where lymphoid leukemia was listed as the underlying cause, and septicemia was a contributing cause. Over the study period, the AAMR for lymphoid leukemia with septicemia was initially stable from 1999 to 2010 (APC: -0.38%), followed by a decline from 2010 to 2017 (APC: -2.85%), and continued to decline from 2017 to 2020 (APC: -6.34%). The highest mortality was observed in males (62.7%), White individuals (86.60%), metropolitan regions (82.6%), and medical facilities (89%).

The cause of septicemia in lymphoid leukemia is multifactorial. Even before chemotherapy begins, individuals with leukemia often have weakened immunity. This is because abnormal leukemic cells crowd the bone marrow, interfering with the formation of normal white blood cells; as a result, these patients are at increased risk for infections caused by a wide range of pathogens [10]. Additionally, treatments used to manage the condition further weaken immune defenses by causing hypogammaglobulinemia. Different therapies are often associated with distinct types of infectious complications [11]. Low levels of immunoglobulins significantly increase the risk of septicemia, both in individuals with early-stage disease and in those receiving traditional alkylating chemotherapy drugs [12]. The reduction in IgG3 and IgG4 immunoglobulin levels is mainly attributed to impairments in both T-cell function and the activity of non-clonal CD5− B cells, contributing significantly to the overall immune deficiency seen in these patients [13]. Furthermore, studies have shown that the length of the disease as well as the advanced stage of the disease are also associated with septicemia [14].

Our study found an overall AAMR of 17,265 per million for lymphoid leukemia with septicemia; the crude rate per million is 3.9 over the study period. This study found that males had a higher AAMR than females. The total number of deaths in males is 10,825 (62.7%) compared to females' 6,440 (37.3%). Prior observational studies have found similar findings, yet the reason behind it remains unclear. Future prospective cohort studies will help assess established molecular, genetic, and clinical prognostic factors in males' and females' mortality differences [15].

Further, the study shows the mortality rate is higher in metropolitan areas, with total deaths of 1,42,679 (82.60%), compared to non-metropolitan areas, with total deaths of 2,998 (17.20%). One of the major reasons behind this data could be that critically ill patients, such as those with lymphoid leukemia and septicemia, are frequently referred to urban tertiary care centers, leading to a higher volume of deaths recorded in metropolitan hospitals compared to rural settings [16]. Also, metropolitan hospitals typically have better infrastructure for data collection and mortality reporting, leading to more accurate death documentation [17].

Medical facilities had the highest mortality (89%) compared to other settings. Unlike solid tumors, hematologic malignancies like leukemia are associated with lower referral rates to hospice. Clinicians often pursue aggressive care longer, delaying end-of-life transition planning [18]. Also, sepsis can cause a rapid, unexpected decline, often prompting emergency hospitalization even if patients were previously managed at home or in long-term care. Race has also shown significant variation in mortality. The White population has the highest mortality rate (86.60%). The disparities in leukemia incidence among the races are not well explained by established risk factors. Additional research focusing on genetic and environmental variations across populations, particularly by leukemia subtype, may help uncover the underlying causes of these diseases [19].

Rising mortality rates among lymphoid leukemia patients with septicemia highlight the urgent need for coordinated care approaches that combine oncology and infectious disease expertise. Future research should look at which patients are most at risk, how treatments affect them over time, and how to prevent serious infections. It's also important to improve healthcare in rural areas, reduce racial differences in lymphoid leukemia treatment, and include infection risk in lymphoid leukemia care plans. Long-term studies following lymphoid leukemia survivors can help us better understand how cancer treatments may increase the risk of dying from infections.

Limitations

This study has several limitations, including potential coding errors in death certificate data and the absence of information on comorbidities and treatment history. Shifts in diagnostic and reporting standards over time may have affected the observed trends. Additionally, the MCD database does not include socioeconomic status, healthcare access, or lifestyle factors, which may have impacted results. The lack of microbiological or clinical data further restricts insight into the nature and severity of septicemia in lymphoid leukemia patients. As a retrospective observational study, it cannot establish causation.

## Conclusions

Based on the retrospective analysis done using data from the CDC MCD database between 1999 and 2020, we observed that the mortality trends in patients with lymphoid leukemia with septicemia had a crude death rate of 3.9 per million population, skewed towards the male, metropolitan, and White population. It is essential to note that while we could see the rising trend of septicemia in lymphoid leukemia patients, the risk factors underlying the variation in gender, race, and geography remain to be explored through longitudinal observational studies. It is also crucial to identify the co-morbidities and other lifestyle factors in patients with lymphoid leukemia to enable prompt treatment if septicemia occurs, which ultimately requires extensive coordination between specialties such as the department of oncology and the department of infectious diseases.
